# Integrated analysis of single-cell and bulk transcriptome reveals hypoxia-induced immunosuppressive microenvironment to predict immunotherapy response in high-grade serous ovarian cancer

**DOI:** 10.3389/fphar.2024.1450751

**Published:** 2024-11-13

**Authors:** Qingshan Chen, Yue Zhang, Chao Wang, Hui Ding, Liqun Chi

**Affiliations:** ^1^ Department of Pharmacy, Shanghai Key Laboratory of Maternal Fetal Medicine, Shanghai Institute of Maternal-Fetal Medicine and Gynecologic Oncology, Shanghai First Maternity and Infant Hospital, School of Medicine, Tongji University, Shanghai, China; ^2^ Department of Gynecology, Shanghai Key Laboratory of Maternal Fetal Medicine, Shanghai Institute of Maternal-Fetal Medicine and Gynecologic Oncology, Shanghai First Maternity and Infant Hospital, School of Medicine, Tongji University, Shanghai, China; ^3^ Department of Pharmacy, Haidian Maternal and Child Health Hospital of Beijing, Beijing, China

**Keywords:** high-grade serous ovarian cancer, hypoxia, immunotherapy, immunosuppressive microenvironment, single-cell analysis

## Abstract

**Background:**

Hypoxia is significantly associated with cancer progression and treatment outcomes. Nevertheless, the precise molecular mechanisms underlying the hypoxia-induced immunosuppressive microenvironment in high-grade serous ovarian cancer (HGSOC) are still not fully understood.

**Methods:**

By analyzing five independent transcriptomic datasets, we investigated the effect of hypoxia on prognosis and tumor microenvironment (TME) in HGSOC. The hypoxia levels and the intercellular communication signaling pathways were studied by using single-cell analysis. Furthermore, the Hypoxia-TME classifier was developed and then validated in the multiple HGSOC datasets. In addition, we also investigated the prognostic significance, genetic variations, signaling pathways, and the potential for immunotherapy benefits in different Hypoxia-TME subgroups.

**Results:**

Hypoxia was identified as a crucial risk factor in HGSOC, and strongly correlated with an immunosuppressive microenvironment characterized by alterations in the composition and distribution of immune cells. Single-cell analysis elucidated the heterogeneity inherent within the TME in HGSOC, and demonstrated an association between the hypoxic TME and fibroblasts as well as macrophages. CellChat analysis identified SPP1-CD44 and CXCL12-CXCR4 as the principal signaling axes through which macrophages and fibroblasts interact with T cells, respectively. Moreover, a personalized Hypoxia-TME classifier was constructed and validated through the integration of the hypoxia (18 genes) and TME (7 immune cells) scores. It was observed that patients in the Hypoxia^low^/TME^high^ subgroup displayed a significantly better prognosis than other subgroups. Different subgroups exhibited unique genomic alterations and variations in signaling pathway differences, including TGF-β and Wnt/β-catenin pathways, which are closely associated with various biological functions. Finally, our results indicated that patients in the Hypoxia^low^/TME^high^ subgroup exhibit a better response to immunotherapy, suggesting the potential utility of the Hypoxia-TME classifier as a new biomarker in HGSOC.

**Conclusion:**

Our study revealed hypoxia-induced immunosuppressive microenvironment, and developed Hypoxia-TME classifier to distinguish the prognosis, immune characteristics, and potential benefits of immunotherapy in HGSOC.

## 1 Introduction

Globally, ovarian cancer is a prevalent gynecological malignancy with the highest mortality rate among all gynecological tumors (Lheureux et al., 2019). As the predominant histological subtype of ovarian cancer, high-grade serous ovarian cancer (HGSOC) accounts for an estimated 70%–80% of all ovarian cancer-related mortality (Siegel et al., 2019; Matulonis et al., 2016). HGSOC patients often present with tumor metastasis and are frequently diagnosed at advanced stages. Surgical resection combined with neoadjuvant chemotherapy is the standard management for patients, most patients will likely chemoresistance and recurrence, and thus the overall survival (OS) rates of patients remain very low (Bohm et al., 2016; Kuroki and Guntupalli, 2020). Therefore, studying the primary factors influencing the survival prognosis of HGSOC and the associated molecular mechanisms will offer novel therapeutic strategies for patients.

Recent research indicates that immunotherapy with immune checkpoint inhibitors can significantly improve clinical outcomes of patients in several cancers, such as melanoma, neuroblastoma, and non-small cell lung cancer (Billan et al., 2020; Reck et al., 2022; Huang and Zappasodi, 2022; Anderson et al., 2022). Multiple clinical trials have provided evidence of the anti-tumor efficacy and safety of immune checkpoint inhibitors in patients with platinum-resistant or recurrent ovarian cancer (Xia et al., 2022; Hamanishi et al., 2021; Lee et al., 2020). For example, the KEYNOTE-100 trial demonstrated that pembrolizumab exhibited anti-tumor efficacy and a well-tolerated safety dose in advanced ovarian cancer patients with recurrence (Lee et al., 2020). The influence of the tumor microenvironment (TME) on tumor prognosis and immunotherapy effectiveness is widely recognized. Nevertheless, malignant tumor cells can evade immune surveillance by utilizing intricate immune evasion mechanisms, which involve immune cell dysfunction, immune checkpoint signaling, and genetic alterations (Mohme et al., 2017; van Weverwijk and de Visser, 2023). Importantly, HGSOC is considered as an immune cold tumor due to limited immune cell infiltration around the tumor site, resulting in reduced responsiveness to immunotherapy in HGSOC patients (Kandalaft et al., 2022). Hence, it is necessary to elucidate the molecular mechanisms that contribute to the immunosuppressive microenvironment in HGSOC, and simultaneously explore novel predictive models and therapeutic strategies.

This study involved a comprehensive investigation that included 807 HGSOC patients from 5 multicenter studies, with the aim of systematically analyzing the cancer hallmarks and immune microenvironment. The results indicated that hypoxia was the primary prognostic risk factor in HGSOC, exhibiting a significant association with the immunosuppressive microenvironment. Subsequently, we investigated the impact of hypoxia on immune cell populations through the utilization of single-cell RNA sequencing (scRNA-seq) analysis. Additionally, we characterized and analyzed cell-cell communication networks. Moreover, we constructed and validated a personalized classifier that integrates hypoxia and immune cells, enhancing risk stratification and predictive precision for patients with HGSOC. Finally, we elucidated the link between the Hypoxia-TME classifier, somatic mutations, and immune characteristics to guide prognosis management and immunotherapy decisions for HGSOC patients.

## 2 Methods

### 2.1 Collection and processing of bulk transcriptome data from patients with HGSOC

In this study, we collected transcriptome data and corresponding clinical information from five public cohorts of HGSOC patients, including RNA-sequencing dataset from The Cancer Genome Atlas-Ovarian Cancer (TCGA-OV) and four microarray datasets (GSE13876, GSE14764, GSE18520, and GSE26712). After excluding patients with incomplete survival information, a total of 807 HGSOC patients were selected for subsequent study (Supplementary Table S1), including TCGA-OV (n = 378), GSE13876 (n = 157), GSE14764 (n = 66), GSE18520 (n = 53), and GSE26712 (n = 153). For RNA-sequencing dataset, raw counts were normalized by transcripts-per-million bases (TPM) and log2 (TPM+1) transformed. Similarly, the microarray data underwent log2 transformation and normalization by using the Robust Multichip Average algorithm. Moreover, the TCGA-OV dataset (training dataset) was utilized to develop Hypoxia-TME classifier for HGSOC patients, while the four microarray datasets were merged into meta-cohort (validation dataset) for independent performance assessment. In this study, we used ComBat algorithm to eliminated the batch effects among four microarray cohorts by *sva* package. The *sva* is a well-known package that can be used to identify, estimate, and remove various sources of variation in high-throughput data (Leek et al., 2012).

### 2.2 Cancer hallmarks, immune cells, and functional enrichment analysis

We collected a total of 29 cancer hallmarks gene sets from the Molecular Signatures Database (MSigDB) (Liberzon et al., 2015). The performances of cancer hallmarks were calculated by single sample gene set enrichment analysis (ssGSEA) algorithm by using GSVA package. In order to investigate the tumor immune microenvironment in HGSOC, we further estimated the relative content of the immune cells by using the ssGSEA algorithm. The gene signatures of 28 tumor-infiltrating immune cells have been identified in previous study (Ru et al., 2019). Moreover, Tumor Immune Dysfunction and Exclusion (TIDE) algorithm was used to study the immune dysfunction and exclusion mechanisms in tumors (Jiang et al., 2018). The TIDE score of HGSOC patients was calculated online to explore the predictive capacity of Hypoxia-TME classifier for immunotherapy outcomes. The Imvigor210 cohort was also employed to predict the therapeutic responses of HGSOC patients to anti-PD-L1 immunotherapy. Additionally, the exome sequencing data of HGSOC was downloaded from TCGA-OV cohort (Cancer Genome Atlas Research, 2011). Tumor mutational burden (TMB) refers to the quantity of non-synonymous mutations found in every million bases (Mb) of the genomic sequence under investigation. The somatic mutations landscape and TMB was analyzed by using the *maftools* package (Mayakonda et al., 2018).

### 2.3 Single-cell RNA sequencing data processing of HGSOC patients

The scRNA-seq data from five HGSOC patients were obtained from the Gene Expression Omnibus (GEO) database (GSE112302) (Geistlinger et al., 2020). The scRNA-seq data were processed using the R package *Seurat*, which included quality control, normalization, dimensionality reduction, clustering, and cell type identification (Hao et al., 2021; Andrews et al., 2021). Cells with a minimum detection of 200 genes and expression in at least three cells were retained. After quality control, the data were normalized, and 2000 highly variable features were identified. Harmony is a tool used for batch effects correction, thereby improving data integration and downstream analysis (Korsunsky et al., 2019). Harmony was applied to remove possible batch effects in the GSE112302 dataset. Subsequently, the top 2,000 variant genes were analyzed using the standard deviation in principal component analysis (PCA). In this study, we selected the top 30 principal components (PCs) for clustering, dimension reduction, and visualization analysis via the application of the uniform manifold approximation and projection (UMAP) method. Cell types were defined according to marker gene expression, and automatic annotation by using *SingleR* package (Aran et al., 2019). The specific marker genes were gathered from both published literature and the CellMarker database (Hu et al., 2023). The differential marker genes between distinct cell populations in the GSE112302 dataset were distinguished through the FindAllMarkers function available in the *Seurat* package. In addition, cell-cell interaction signaling pathways was analyzed by using *CellChat* package, and the “CellChatDB.human” database as a reference for ligand-receptor interactions.

### 2.4 Development of hypoxia signature, TME signature and Hypoxia-TME classifier

To construct the hypoxia signature for patients with HGSOC, we screened for hypoxia-related genes using univariate Cox regression analysis and Least Absolute Shrinkage and Selection Operator (LASSO) regression method. Through dimension reduction screening, significant prognostic genes were selected and used to construct the hypoxia-related gene signature. The hypoxia score was calculated by weighting the expression levels of the candidate genes with the LASSO coefficients. The hypoxia subgroups were determined based on the mean value of hypoxia score in each cohort. Then, the differential marker genes of TME-related cells were obtained from scRNA-seq analysis. Based on theses marker genes, the relative content of TME-related cells was estimated by using the ssGSEA algorithm. Similarly, the TME score was also calculated from the TME-related cells selected by the LASSO method. According to the mean value of TME score, patients were classified into the TME-high and TME-low subgroups in each cohort. Furthermore, we combined the hypoxia and TME scores to develop the Hypoxia-TME classifier. Patients were further categorized into the following subgroups: Hypoxia^low^/TME^high^, mixed (Hypoxia^low^/TME^low^ and Hypoxia^high^/TME^high^) and Hypoxia^high^/TME^low^. The efficacy of the Hypoxia-TME classifier in predicting clinical outcomes was assessed through Kaplan-Meier (K-M) survival analysis and receiver operating characteristic (ROC) analysis in each HGSOC cohorts.

### 2.5 Immunofluorescence staining

Tumor tissues of 3 HGSOC patients were obtained from Shanghai First Maternity and Infant Hospital (Shanghai, China). All patients have signed informed consent admitted to hospital. Immunofluorescence staining was described in our previous study (Wei et al., 2018). Briefly, tumor tissues were deparaffinized and blocked with 3% bovine serum albumin. The sections were labeled with primary antibodies COL1A1 (ab34710, Abcam; 1:150 dilution) and PD-L1 (ab205921, Abcam; 1:200 dilution) overnight and subsequently incubated with antibody HIF-1α (ab8366, Abcam, 1:150 dilution) for double staining, respectively. Next, the fluorescent secondary conjugated Alexa Fluor-488 and Alexa Fluor-594 were incubated for 2 h. After washing, the cell nuclei were counterstained with 4′,6-diamidino-2-phenylindole (DAPI).

### 2.6 Statistical analysis

The statistical analysis was performed using R software (v4.2.2). Survival analysis was conducted using the K-M curves and the log-rank test implemented in the R packages *survminer* and *survival*. We performed statistical comparisons between two groups using the Student’s t-test, and comparisons among three groups using one-way analysis of variance (ANOVA) analysis. Benjamini-Hochberg (BH) correction was applied for multiple hypothesis testing. Statistical significance was defined as p < 0.05.

## 3 Results

### 3.1 Hypoxia was identified as an important risk factor in HGSOC

**FIGURE 1:**
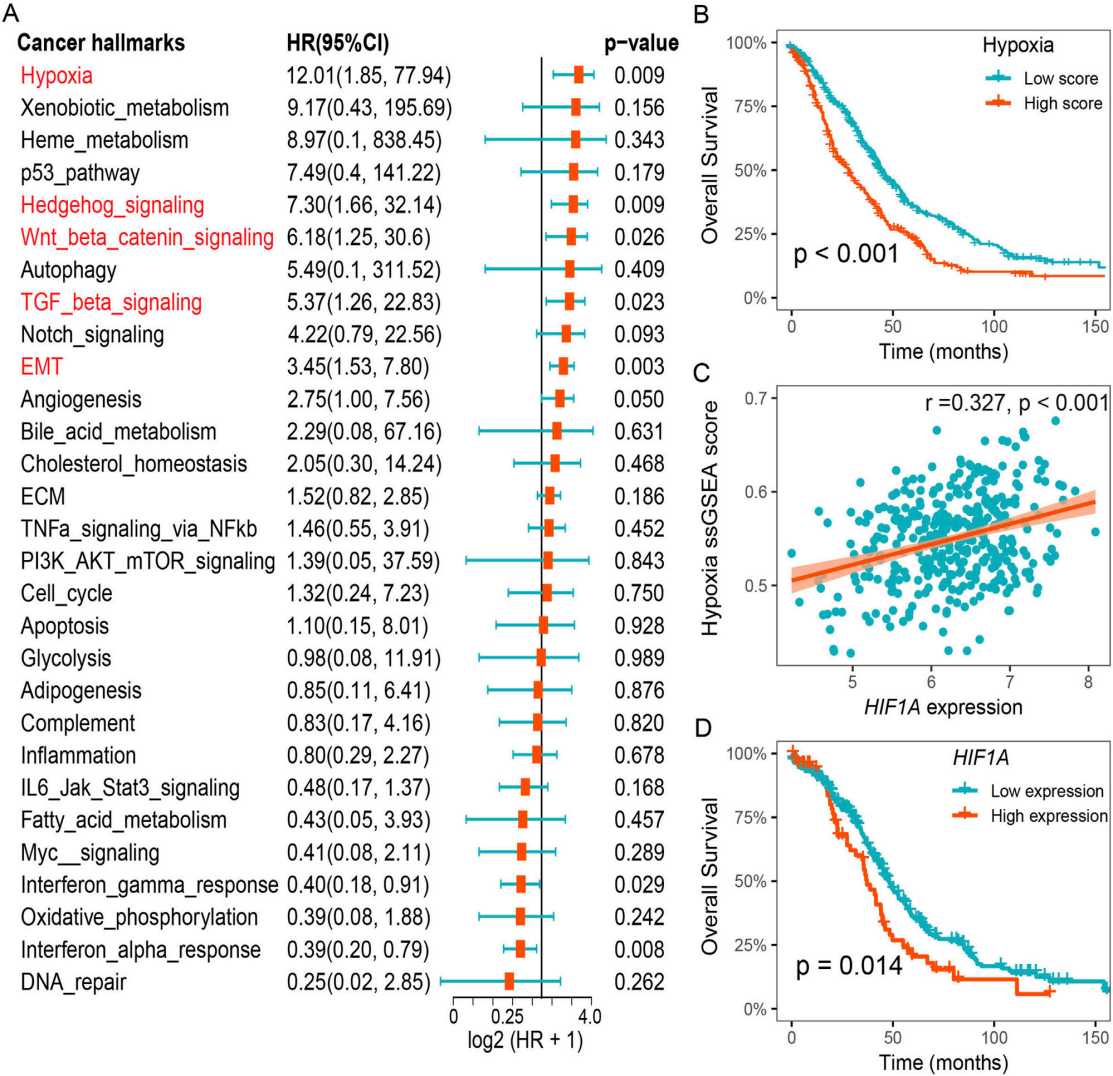
Hypoxia was identified as an important risk factor for prognosis in HGSOC. **(A)** Meta analysis of cancer hallmarks for prognosis in HGSOC cohorts. **(B)** Survival analysis of the hypoxia-related ssGSEA score. **(C)** Correlation between hypoxia ssGSEA score and *HIF1A* expression. **(D)** Survival analysis of the *HIF1A* expression in the TCGA-OV cohort.

To identify significant risk factors in HGSOC, we calculated the prognostic value of cancer hallmarks using the gene expression profiles of 807 patients across 5 HGSOC cohorts (Supplementary Table S2). We then used meta-analysis to integrate overall prognostic value of each cancer hallmarks. The results revealed five cancer hallmarks, including hypoxia, Hedgehog signaling, Wnt/β-catenin signaling, TGF-β signaling, and epithelial-mesenchymal transition (EMT), all of which were identified as significant risk factors (Figure 1A). After adjusting for multiple hypothesis testing, hypoxia emerged as the primary risk factor for OS among all cancer hallmarks in HGSOC. Survival analysis further suggested that HGSOC patients with lower hypoxia score had a better OS compared to patients with higher hypoxia score (Figure 1B). Furthermore, we found that a notable positive relationship between the expression of *HIF1A* and the hypoxia ssGSEA score, and lower expression of *HIF1A* was also associated with better survival outcomes for patients in the TCGA-OV cohort (Figures 1C, D). These results suggest that hypoxia was a dominant risk factor for prognosis in HGSOC.

### 3.2 Hypoxia is associated with immunosuppressive microenvironment

**FIGURE 2:**
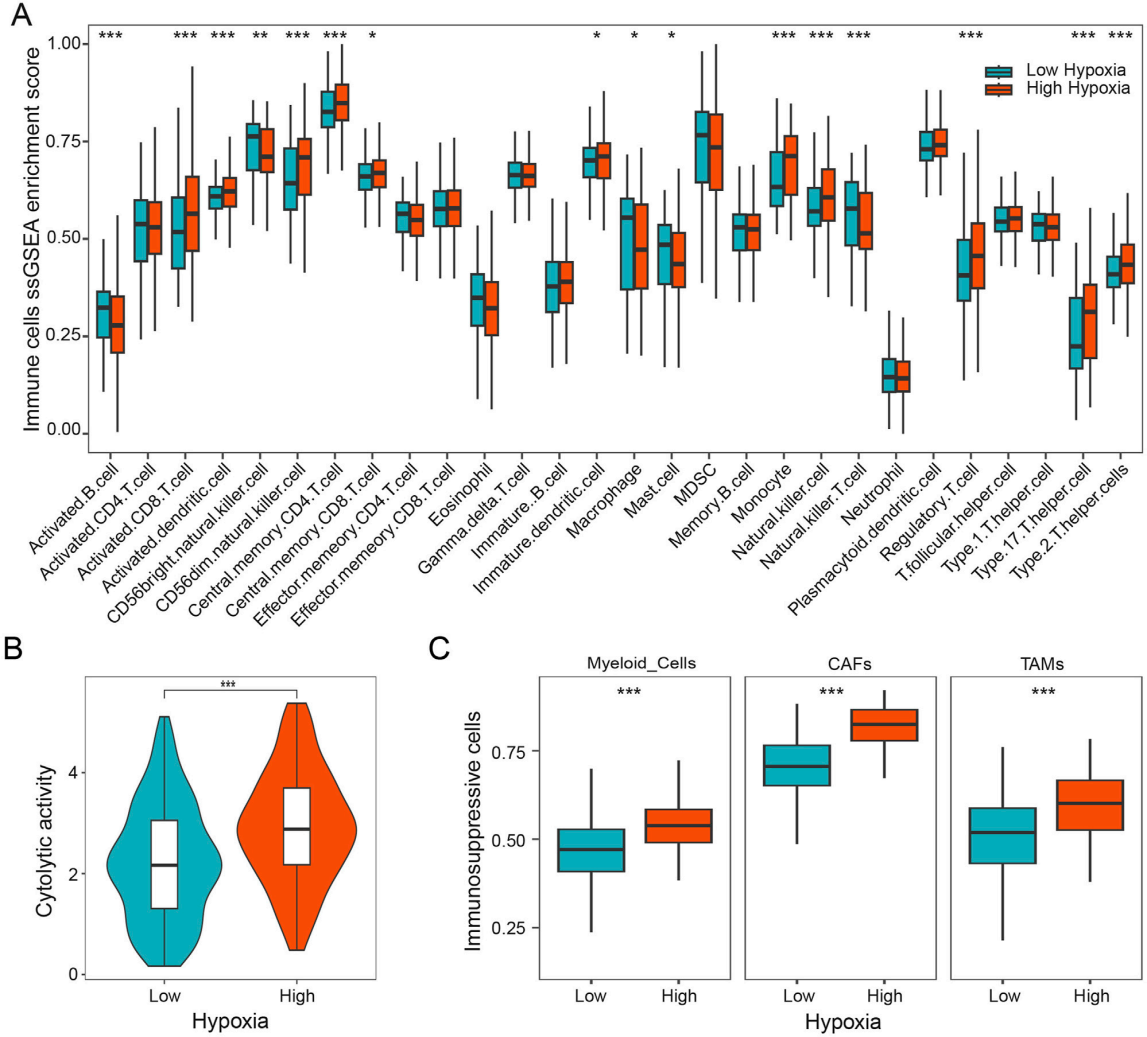
Hypoxia is associated with immunosuppressive microenvironment. **(A)** Infiltration 28 immune cell types in HGSOC with different hypoxia groups. **(B)** The cytolytic activity of patients in different hypoxia groups. **(C)** The relative content of myeloid cells, CAFs, and TAMs in different hypoxia groups. *, p < 0.05; **, p < 0.01; ***, p < 0.001.

To study the role of hypoxia on tumor immune microenvironment, we characterized the composition of immune cells in patients with HGSOC by using the ssGSEA method. Our results showed that the distribution of immune cells exhibits markedly difference between different hypoxia subgroups. As shown in Figure 2A, we found that activated CD8 T cells, monocyte, regulatory T cells, and Th 17 cells were more abundant in the patients with higher hypoxia score, while activated B cells, macrophage, and natural killer T cells were more abundant in the patients with lower hypoxia score. Furthermore, the cytolytic activity was calculated, which was found to be notably increased in HGSOC patients with high hypoxia score (Figure 2B). Subsequently, we investigated three immunosuppressive cells known to restrict T cell infiltration, specifically myeloid cells, cancer-associated fibroblasts (CAFs), and tumor-associated macrophages (TAMs). Our findings demonstrated a significant increase in myeloid cells, CAFs, and TAMs in patients with high hypoxia score (Figure 2C). Additionally, the expression of *HIF1A* was significantly positively linked to the expressions of *FOXP3*, *CD163*, and *COL1A1* (Supplementary Figure S1). These results indicate that hypoxia is associated with immunosuppressive microenvironment in HGSOC.

### 3.3 The effect of hypoxia in the TME through scRNA-seq analysis

**FIGURE 3:**
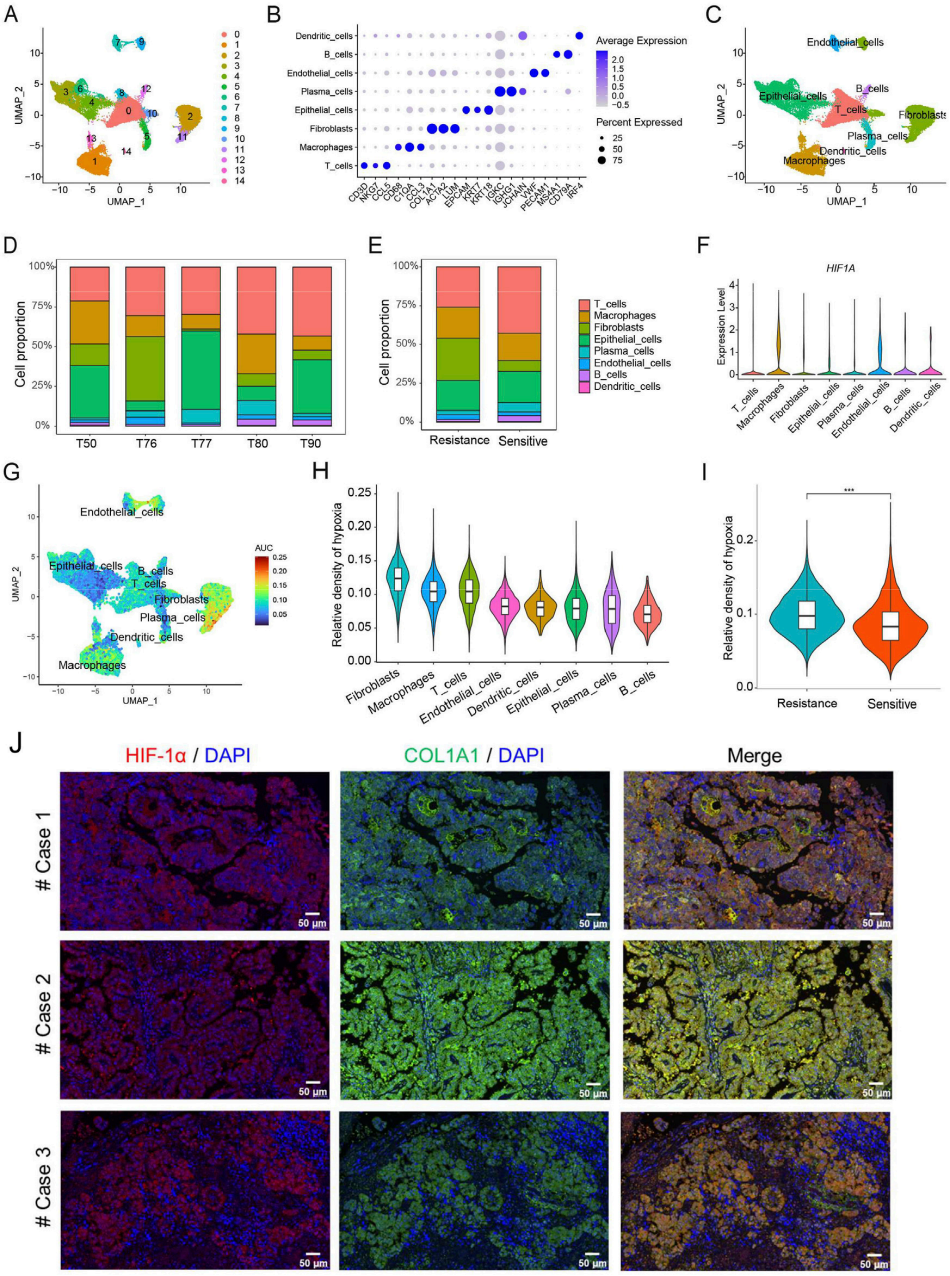
Analysis of hypoxia in the TME at the level of single-cell RNA sequencing. **(A)** The UMAP plot displays 15 cell types in HGSOC. **(B)** The specific marker genes in each cell types. **(C)** Eight cell types annotation. **(D)** Proportion of eight cell types. **(E)** Proportion of eight cell types in chemotherapy-sensitive and resistant patients. **(F)** Expression levels of *HIF-1A* in eight cell types. **(G)** Hypoxia score across single cells. **(H)** Hypoxia score among eight distinct cell types. **(I)** The differences of hypoxia score between chemotherapy-sensitive and resistant patients. **(J)** Immunofluorescence staining of HIF-1α and COL1A1 in HGSOC patients. ***, p < 0.001.

We further analyzed scRNA-seq data obtained from five patients with HGSOC. After quality control, 51,643 cells were clustered into 15 subpopulations and annotated into eight cell types, including T cells, macrophages, fibroblasts, epithelial cells, plasma cells, endothelial cells, B cells, and dendritic cells (Figures 3A–C). The specific marker genes of eight cell types in HGSOC have been identified. Moreover, our results clearly showed that the proportion of eight cell types among patients is different, which reflects the heterogeneity of the immune microenvironment in HGSOC patients (Figure 3D). The chemotherapy-resistant group exhibited a higher abundance of fibroblasts and macrophages, whereas the chemotherapy-sensitive group showed a higher abundance of T cells (Figure 3E). At the single-cell level, we found that the expression of *HIF1A* was mainly present in macrophages, fibroblasts, and endothelial cells (Figure 3F). Additionally, we calculated the hypoxia score for the eight cell types in HGSOC, revealing that fibroblasts exhibited the highest hypoxia score compared to the other seven cell types (Figures 3G, H). Furthermore, we observed that chemotherapy-resistant patients displayed a higher hypoxia score compared to chemotherapy-sensitive patients (Figure 3I). In HGSOC patients, the results of immunofluorescence staining also demonstrated that the expression of HIF-1α was mainly present in fibroblasts (Figure 3J). These findings further indicate that hypoxia induces immunosuppressive microenvironment in HGSOC, potentially involving fibroblasts and macrophages.

### 3.4 Cell-cell communications in the progression of HGSOC

**FIGURE 4:**
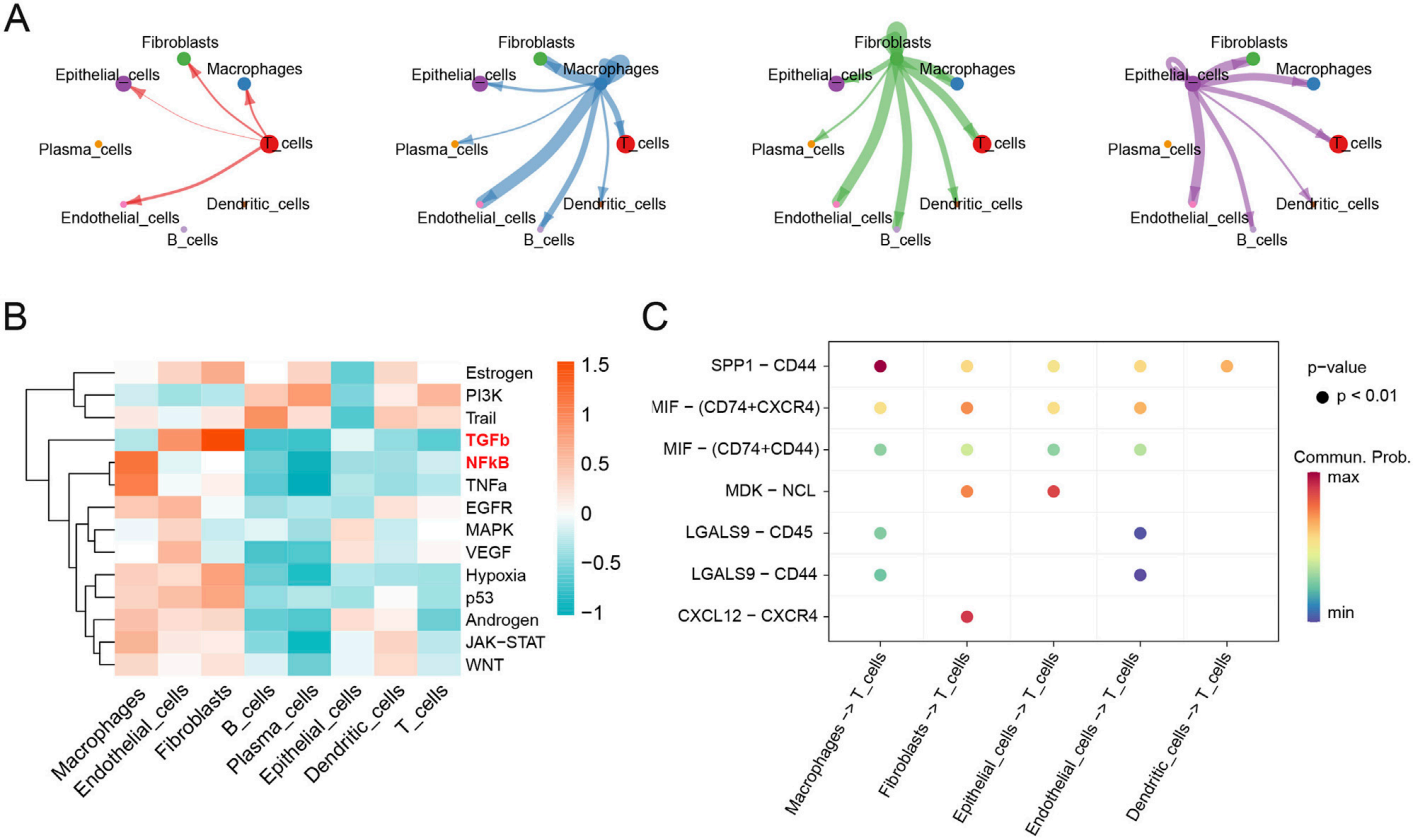
Cell-cell communications in the progression of HGSOC. **(A)** Cell-cell communications; **(B)** Mean pathway activity scores; **(C)** Ligand-receptor pairs between T cells and other cell groups.

CellChat analysis was performed to explore cell-cell communications involved in the progression of HGSOC. The interaction numbers and strength between eight cell types were depicted in detail (Supplementary Figure S2A). Fibroblasts, macrophages, epithelial cells, and T cells exhibited stronger interaction numbers and strength with other cell types in HGSOC (Figure 4A). Then, specific pathways were identified in different cell types, and TGF-β, and NF-κB signaling pathways were mainly activated in the fibroblasts and macrophages, respectively (Figure 4B). Furthermore, we specifically analyzed the ligand-receptor pathways that potentially regulate intercellular communications between T cells and other cells. It was found that macrophages, fibroblasts and epithelial cells exhibited the strongest interactions with T cells (Figure 4C). Notably, macrophages communicated with T cells via the SPP1-CD44 signaling pathway, while fibroblasts interacted with T cells through the CXCL12-CXCR4 and MIF-(CD74 + CXCR4) signaling pathways. Furthermore, fibroblasts communicated with epithelial cells via the MDK-NCL pathway (Supplementary Figure S2B). These results indicate that intercellular communication is crucial in the progression of HGSOC.

### 3.5 Development of the Hypoxia-TME classifier improved prognostic evaluation

**FIGURE 5:**
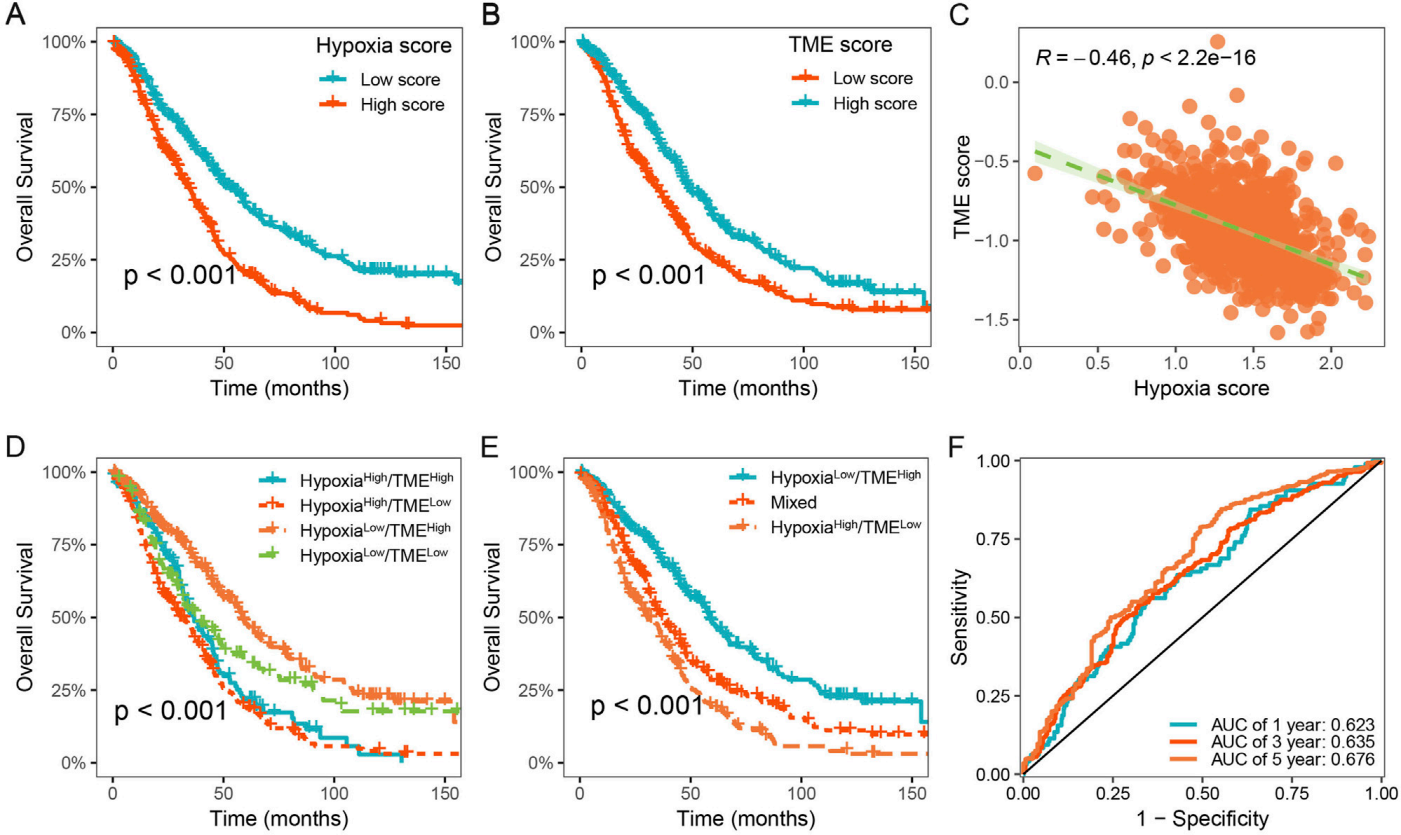
Construction of the Hypoxia-TME classifier improved prognostic assessment. **(A)** Survival analysis of the Hypoxia score. **(B)** Survival analysis of the TME score. **(C)** Correlation between the Hypoxia and TME scores. **(D, E)** Survival analysis of the Hypoxia-TME classifier. **(F)** ROC analysis of the Hypoxia-TME classifier.

To characterize the hypoxic immune microenvironment in HGSOC, we characterized immune cells based on the differentially expressed markers derived from scRNA-seq data (Supplementary Table S3). We investigated the prognostic values of 179 hypoxia-related genes using univariate Cox regression analysis and meta-analysis. We found that 35 hypoxia-related genes were significantly associated with OS in HGSOC (p < 0.05; Supplementary Table S4). Subsequently, hypoxia score and TME score were developed using LASSO Cox regression analysis, respectively (Supplementary Table S5). HGSOC patients were divided into low- and high-hypoxia groups based on the mean value of the hypoxia score. It was observed that HGSOC patients with high hypoxia score exhibited shorter OS time than those with low hypoxia score (Figure 5A). Similarly, a significant difference between low- and high-TME groups was also observed (Figure 5B). In addition, there was a significant negative correlation between the hypoxia and TME score (Figure 5C).

Based on the results above, we considered whether the hypoxia and TME score could be combined to further stratify HGSOC patients. Consequently, we constructed the Hypoxia-TME classifier by integrating the hypoxia and TME score, which allowed the patients to be divided into four subgroups: Hypoxia^low^/TME^high^, Hypoxia^low^/TME^low^, Hypoxia^high^/TME^high^, and Hypoxia^high^/TME^low^. Our results showed that Hypoxia-TME classifier exhibited a significant prognostic difference in patient with HGSOC (Figure 5D). It was observed that patients in the Hypoxia^low^/TME^high^ subgroup exhibited the most favorable prognosis. In addition, the prognosis in the Hypoxia^low^/TME^low^ and Hypoxia^high^/TME^high^ subgroups showed less divergent, and thus we merged these two subgroups (Figure 5E). Finally, it was showed that the area under the curve (AUC) of the Hypoxia-TME classifier were 0.623, 0.635, and 0.676 for 1-, 3-, and 5- years OS, respectively (Figure 5F). These results indicate that Hypoxia-TME classifier improves the accuracy of prognostic prediction for patients with HGSOC.

### 3.6 Validation and evaluation of the Hypoxia-TME classifier in multicenter studies

**FIGURE 6:**
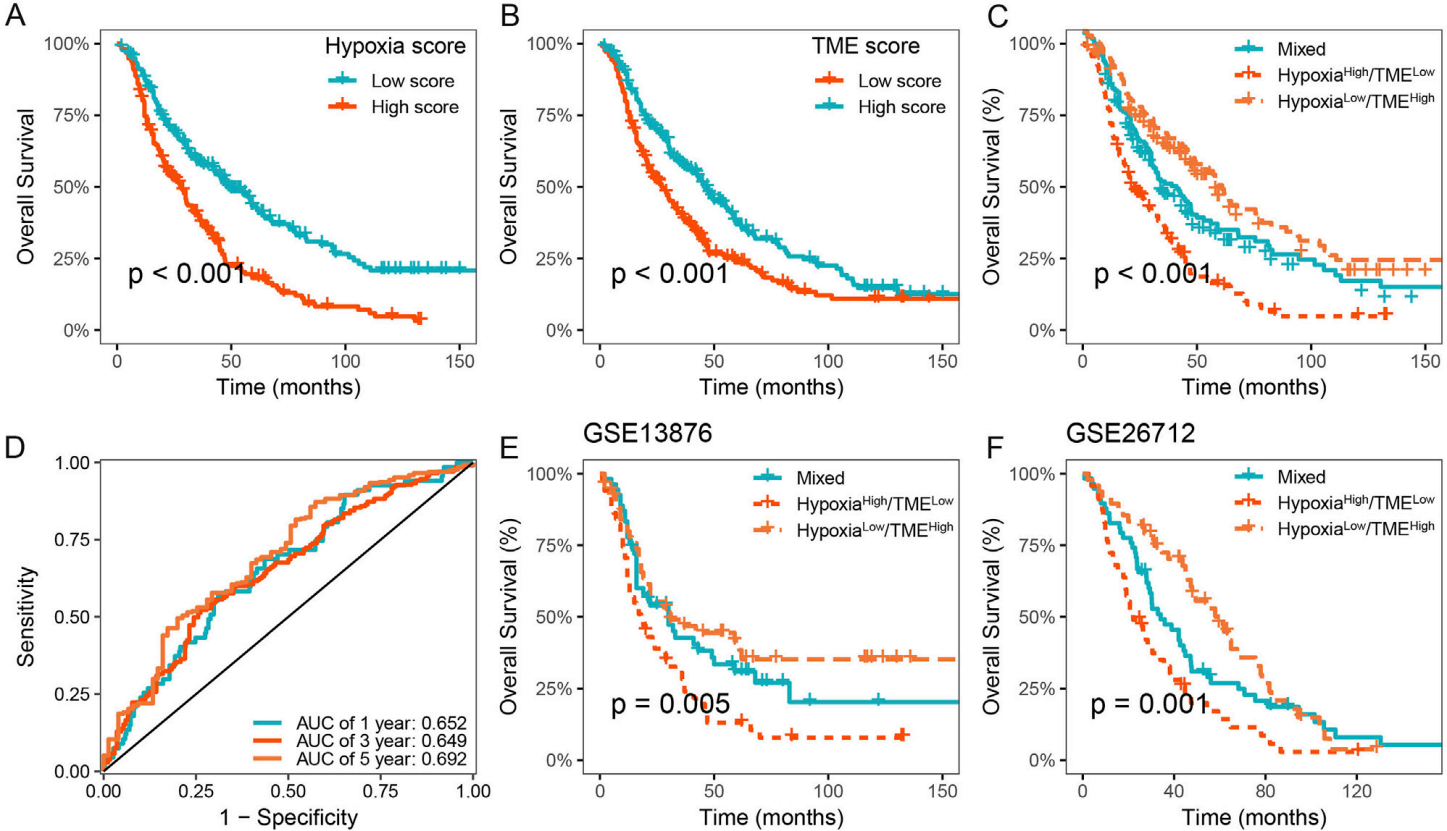
Validation of the Hypoxia-TME classifier in multicenter studies. **(A)** Survival analysis of the Hypoxia score in the meta cohort. **(B)** Survival analysis of the TME score in the meta cohort. **(C)** Survival analysis of the Hypoxia-TME classifier in the meta cohort. **(D)** ROC analysis of the Hypoxia-TME classifier in the meta cohort. **(E)** Survival analysis of the Hypoxia-TME classifier in the GSE13876. **(F)** Survival analysis of the Hypoxia-TME classifier in the GSE26712.

We further validated the prognostic value of the Hypoxia-TME classifier in multicenter studies. Our results also demonstrated a poorer prognosis in patients with high hypoxia score or low TME score in the meta-cohort (Figures 6A, B). Similarly, the Hypoxia-TME subgroups showed a distinct prognosis, and patients in the Hypoxia^low^/TME^high^ subgroup had the most favorable prognosis compared to patients from the other subgroups (Figure 6C). As shown in Figure 6D, the ROC analysis depicted that the AUC of the Hypoxia-TME classifier were 0.652, 0.649, and 0.692 for the 1-, 3-, and 5-year OS rates, respectively. Moreover, similar clinical outcomes were also observed in the GSE13876, and GSE26712 cohorts (Figures 6E, F). HGSOC patients with Hypoxia^low^/TME^high^ had a longer survival time compared to other patients. Lastly, the univariate and multivariate Cox analysis indicated that the Hypoxia-TME classifier was the independent clinical factor in the TCGA-OV cohort (Table 1). These results demonstrated the prognostic value of the Hypoxia-TME classifier in multicenter studies.

**TABLE 1:** Univariate and multivariate Cox regression analyses of Hypoxia-TME classifier.

Variables	Univariate analysis		Multivariate analysis	
	HR (95% CI)	p	HR (95% CI)	p
Stage				
Stage II	1		1	
Stage III	2.440 (1.002–5.941)	0.050	2.082 (0.840–5.163)	0.114
Stage IV	2.952 (1.164–7.483)	0.023	2.664 (1.030–6.890)	0.043
Grade				
G2	1		1	
G3	1.232 (0.832–1.823)	0.297	1.145 (0.768–1.709)	0.506
G4	1.851 (0.251–13.634)	0.546	2.567 (0.344–19.17)	0.358
Hypoxia-TME classifier				
Hypoxia^Low^/TME^High^	1		1	
Mixed	1.566 (1.142–2.146)	0.005	1.631 (1.182–2.249)	0.003
Hypoxia^High^/TME^Low^	2.009 (1.455–2.774)	<0.001	2.050 (1.474–2.851)	<0.001

### 3.7 Molecular characteristics among different Hypoxia-TME subgroups

**FIGURE 7:**
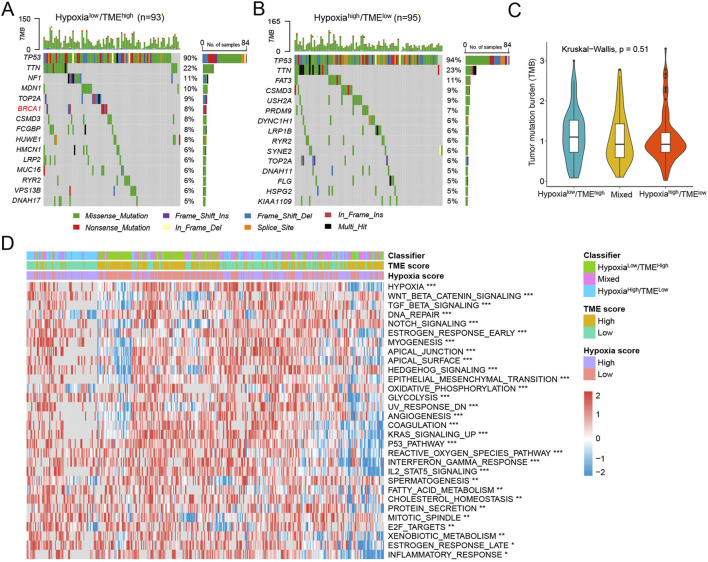
Molecular characteristics in different Hypoxia-TME subgroups. **(A)** Oncoplot of 15 mutated genes in the Hypoxia^low^/TME^high^ group. **(B)** Oncoplot of 15 mutated genes in the Hypoxia^high^/TME^low^ group. **(C)** TMB among Hypoxia-TME classifier subgroups. **(D)** Heatmap of signaling pathways in different Hypoxia-TME subgroups.

In the TCGA-OV cohort, we examined the association between the tumor somatic changes and the Hypoxia-TME classifier. It was found that 420 (96.33%) of 436 HGSOC patients had mutations, with *TP53* being the most frequently mutated gene. We separately analyzed the top 15 genes with the highest mutation frequencies in the different Hypoxia-TME subgroups. Specifically, the Hypoxia^low^/TME^high^ patients exhibited the highest rates of mutation for *TP53* (90%), *TTN* (22%), and *NF1* (11%), while the Hypoxia^high^/TME^low^ patients had the highest mutation frequencies for *TP53* (94%), *TTN* (23%), and *FAT3* (11%) (Figures 7A, B). In addition, we also found that *BRCA1* was the differentially mutated gene in the Hypoxia^low^/TME^high^ subgroup. Since TMB is a potential biomarker of immunotherapy, the TMB score was also calculated for patients. There was no notable difference in TMB across Hypoxia-TME subgroups (Figure 7C). Subsequently, cancer-related molecular pathways were analyzed to study the underlying mechanism among different Hypoxia-TME subgroups. The results clearly showed that distinct patterns of tumor proliferation and immune response among various subgroups (Figure 7D). Specially, the Hypoxia^high^/TME^low^ subgroup were enriched in DNA repair, TGF-β, EMT, and angiogenesis, whereas the Hypoxia^low^/TME^high^ were enriched in IFN-γ response, and inflammatory response.

### 3.8 Prediction of immunotherapy benefit based on the Hypoxia-TME classifier

**FIGURE 8:**
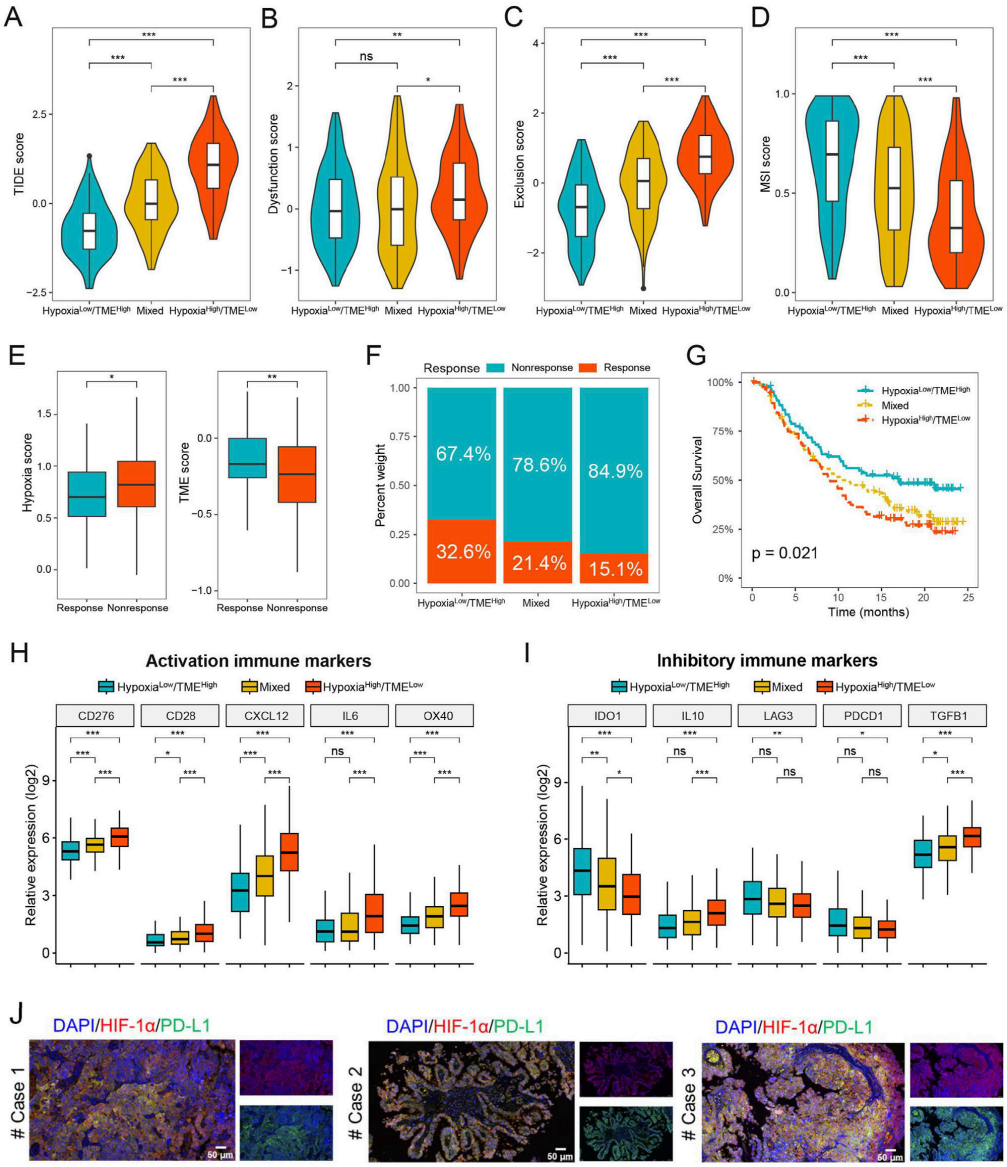
Prediction of immunotherapy benefit based on Hypoxia-TME classifier. **(A–D)** TIDE, T-cell dysfunction and exclusion, and MSI score in different Hypoxia-TME subgroups. **(E)** Hypoxia and TME score in the IMvigor210 cohort. **(F)** The different percentages of anti-PD-L1 immunotherapy in different Hypoxia-TME subgroups. **(G)** Survival analysis of the Hypoxia-TME classifier in the IMvigor210 cohort. **(H, I)** The expression of activation and inhibitory immune markers in different Hypoxia-TME subgroups. **(J)** Immunofluorescence staining of HIF-1α and PD-L1 in HGSOC patients. *, p < 0.05; **, p < 0.01; ***, p < 0.001; ns, not significant.

We further evaluated the possible therapeutic effectiveness of immunotherapy in the different Hypoxia-TME subgroups. The findings showed that the Hypoxia^low^/TME^high^ subgroup had lower TIDE score compared to the Hypoxia^high^/TME^low^ subgroup, indicating that Hypoxia^low^/TME^high^ patients could more benefit from immunotherapy than patients in the Hypoxia^high^/TME^low^ subgroup (Figure 8A). Additionally, the Hypoxia^high^/TME^low^ subgroup had higher T-cell dysfunction and exclusion score, whereas the Hypoxia^low^/TME^high^ subgroup exhibited a higher microsatellite instability score (Figures 8B–D). Furthermore, we assessed the prognostic value of Hypoxia-TME classifier in the anti-PD-L1 immunotherapy cohort IMvigor210. As shown in Figure 8E, patients responding to PD-L1 immunotherapy showed lower hypoxia score or higher TME score, respectively. In addition, the Hypoxia^low^/TME^high^ subgroup had the highest percentage (32.6%) of patients showing therapeutic response and better prognosis, whereas the Hypoxia^high^/TME^low^ subgroup had a response rate of only 15.1% (Figures 8F–G). Hypoxia^high^/TME^low^ subgroup generally exhibited higher expression levels of activation immune markers compared to the Hypoxia^low^/TME^high^ subgroup (Figure 8H). However, it was noted that the expression of *IDO1*, *LAG3* and *PDCD1* were significantly increased in the Hypoxia^low^/TME^high^ subgroup than Hypoxia^high^/TME^low^ subgroup (Figure 8I). We also found that hypoxia can induce the expression of PD-L1 and thereby immune evasion in HGSOC tumor tissues (Figure 8J). These findings suggest that the Hypoxia-TME classifier may be a novel therapeutic biomarker for identifying patients who would benefit from immunotherapy.

## 4 Discussion

HGSOC is characterized by genomic instability, which causes heterogeneity and results in different molecular subtypes, making it challenging to determine effective clinical treatments. Therefore, further study is required to enhance our insights into the molecular mechanisms in HGSOC, aiming to develop new strategies for prognosis and treatment. In this study, by integrating RNA sequencing transcriptome data from 807 HGSOC patients, hypoxia was identified as the most critical risk factor for ovarian cancer. Furthermore, scRNA-seq analysis revealed that the primary cell types in HGSOC include epithelial cells, T cells, macrophages, and fibroblasts, with fibroblasts and macrophages exhibiting higher levels of hypoxia. Currently, several studies have used scRNA-seq analysis to characterize different cell types and therapeutic targets in ovarian cancer and HGSOC (Hu et al., 2020; Wang et al., 2022; Liang et al., 2021). These studies indicate that T cells, fibroblasts, and macrophages are the most important cells in HGSOC, and they are significantly linked to the progression and treatment of HGSOC, which are similar to our results. In addition, a distinct S100A9^+^ tumor cell subtype was identified in both primary and metastatic sites, which is strongly associated with poor prognosis (Xu et al., 2024). Moreover, our cell-cell interaction analysis revealed that the CXCL12-CXCR4 signaling pathway mediated fibroblast-T cell interactions, while the SPP1-CD44 signaling pathway played a major role in macrophage-T cell interactions. These findings indicate that targeting the hypoxia-induced immunosuppressive microenvironment, in combination with immunotherapy, provides new targets and biomarkers for clinical treatment of HGSOC.

Hypoxia represents a crucial characteristic of the solid tumors, which is associated with tumor invasiveness, chemotherapy resistance, and prognosis (Wang et al., 2021). In hypoxic conditions, tumor cells undergo adaptive changes in their metabolic pathways, promoting angiogenesis and invasive growth (Arner and Rathmell, 2023). Furthermore, the hypoxia can impact immune cell function, suppress immune responses, and thus facilitate tumor immune escape (Chen et al., 2023). Hypoxia can inhibit the activity and function of T cells, thereby impairing their potential to eliminate tumor cells. Several studies have demonstrated an association between hypoxia and poor prognosis in ovarian cancer (Wei et al., 2021; Shimogai et al., 2008; Alharbi et al., 2021). Elevated levels of tissue hypoxia correlate with tumor malignancy, recurrence rate, and reduced survival rates. Hence, hypoxia emerges as a potential biomarker for prognostic evaluation in patients with ovarian cancer. In this study, through meta-analysis and integration of multiple HGSOC transcriptome datasets, our results also revealed that patients with high hypoxia scores exhibited a poorer prognosis compared to those with low hypoxia scores. Additionally, the hypoxia-related gene *HIF1A* displayed similar result. These findings imply that targeting hypoxia may improve the clinical treatment outcomes and extend overall survival time of patients with HGSOC.

We then conducted a detailed analysis of the hypoxic TME in HGSOC. Hypoxia is strongly correlated with an immunosuppressive microenvironment, characterized by elevated levels of immunosuppressive cells, including TAMs and CAFs. In this study, we found that the expression of *HIF1A* exhibits a significant positive association with *CD163*, *COL1A1*, and *FOXP3*, suggesting that hypoxia potentially promotes the accumulation of M2 macrophages, fibroblasts, and regulatory T cells. Single-cell analysis further confirmed that the hypoxia-induced immunosuppressive microenvironment is primarily associated with fibroblasts and macrophages, and that hypoxia is related to chemotherapy resistance in HGSOC. CAFs have the ability to secrete various cytokines, including CXCL12, IL-6, and CCL2, which can influence the chemotaxis and function of T cells (Bu et al., 2019). CXCL12 can attract T cells to the vicinity of CAFs, thereby suppressing T cells activation and proliferation. Research has demonstrated that CXCL12β can promote fibroblast heterogeneity and induce an immunosuppressive microenvironment in HGSOC (Givel et al., 2018). Through cell-cell interaction analysis, we discovered that fibroblasts engage in interactions with T cells through the CXCL12-CXCR4 signaling pathway, while macrophages interact with T cells by the SPP1-CD44 signaling pathway. SPP1, through its binding to CD44, can regulate T cell migration and infiltration. Previous studies have identified SPP1 as a biomarker for prognosis in ovarian cancer, which associated with enhanced immune cell infiltration (Gao et al., 2022). These findings highlight the crucial involvement of the CXCL12-CXCR4 and SPP1-CD44 signaling pathways in the hypoxic microenvironment of HGSOC, thereby representing promising targets for therapeutic interventions in HGSOC.

The risk of ovarian malignancy algorithm (ROMA) is a predictive tool that can evaluate the probability and prognosis of epithelial ovarian cancer, calculated by combining the measured values of CA125 and HE4 levels in the blood (Kim et al., 2019). Currently, a variety of gene signatures have been identified as predictive tools for determining the prognosis of patients with ovarian cancer (Wei et al., 2021; Konecny et al., 2016; Bonome et al., 2008). Given the considerable heterogeneity of HGSOC, gene signatures from the single dimension are insufficient to accurately reflect the physiological state of the disease. Thus, it is crucial to identify new biomarkers from multiple dimensions to improve predictive accuracy. Given the pivotal importance of hypoxia and TME as prognostic factors in HGSOC, this study utilized scRNA-seq analysis of immune cells to develop and validate a novel Hypoxia-TME classifier for the prognostic assessment of HGSOC patients. Based on hypoxia score and TME score, patients were categorized into four subgroups: Hypoxia^low^/TME^high^, Hypoxial^low^/TME^low^, Hypoxial^high^/TME^high^, and Hypoxial^high^/TME^low^. The Hypoxia-TME classifier effectively provided significant prognostic stratification for HGSOC patients in multiple independent datasets. Specifically, compared to other subgroups, the subgroup with low hypoxia and high TME (Hypoxia^low^/TME^high^) demonstrated the most favorable survival prognosis. Furthermore, our results showed that the Hypoxia-TME classifier was an independent factor for HGSOC patients through multivariate Cox regression analysis, which can improve prognostic assessment and risk stratification. In addition, racial differences could play a significant role in determining the outcome for patients with HGSOC. Several studies indicated that ovarian cancer patients from different racial exhibit differences in clinical presentation, pathological characteristics, treatment response, and survival rates (Peres et al., 2021; Peres et al., 2022; Sarink et al., 2020).

The predictive capacity of the Hypoxia-TME classifier for the effectiveness of immunotherapy was further explored. We found that Hypoxia^high^/TME^low^ subgroup had more T-cell dysfunction and exclusion, whereas Hypoxia^low^/TME^high^ subgroup showed a greater response rate to immunotherapy. In addition, immune checkpoint-associated biomarkers can also be utilized for predicting the outcomes of immunotherapy treatments. Our results showed that the expression of immune checkpoint genes, including *IDO1*, *LAG3*, and *PDCD1*, had significant differences among Hypoxia-TME subgroups, indicating that different subgroups may have different response rates to immunotherapy. Finally, our investigation focused on the predictive efficacy of the Hypoxia-TME classifier in the IMvigor210 immunotherapy dataset. It was observed that the Hypoxia^low^/TME^high^ subgroup exhibited enhanced immunotherapy response and the most favorable clinical prognosis compared to other subgroups. These findings indicate that the Hypoxia-TME classifier has the capability to identify patients who will benefit from immunotherapy, highlighting its potential utility as an emergent biomarker for immunotherapeutic interventions in HGSOC patients.

Through scRNA-Seq and bulk RNA-Seq analysis of HGSOC patients, the Hypoxia-TME classifier was constructed and subsequently validated across multiple independent datasets. However, there are still some issues that need to be addressed when Hypoxia-TME classifiers are applied to clinical practice. Firstly, it is important to ensure the reliability and accuracy of the classifiers, further evaluation would benefit from the collection of patient samples in real-world. Secondly, our classifier was based on gene expression levels, and more clinical samples are needed to further validate at the protein level. Thirdly, although this study employed a range of bioinformatics methods to develop the Hypoxia-TME classifier, additional functional experiments are required to explore its roles and potential molecular mechanisms *in vivo*. Therefore, future studies should address these limitations.

## 5 Conclusion

This study revealed hypoxia-induced immunosuppressive microenvironment at the single-cell level, and developed a new Hypoxia-TME classifier that contributes to enhanced prognostic prediction capabilities for patients with HGSOC.

## Data Availability

The original contributions presented in the study are included in the article/Supplementary Material, further inquiries can be directed to the corresponding author.
