# Supplementary material for: Integrated analysis of single-cell and bulk transcriptome reveals hypoxia-induced immunosuppressive microenvironment to predict immunotherapy response in high-grade serous ovarian cancer
Source: Front Pharmacol. 2024 Nov 13;15:1450751. doi: 10.3389/fphar.2024.1450751 (PMC11598517; doi:10.3389/fphar.2024.1450751)
Supplement: Supplementary file 1 [file DataSheet1.zip › Figure s1 - s2.DOCX]

Supplementary Material


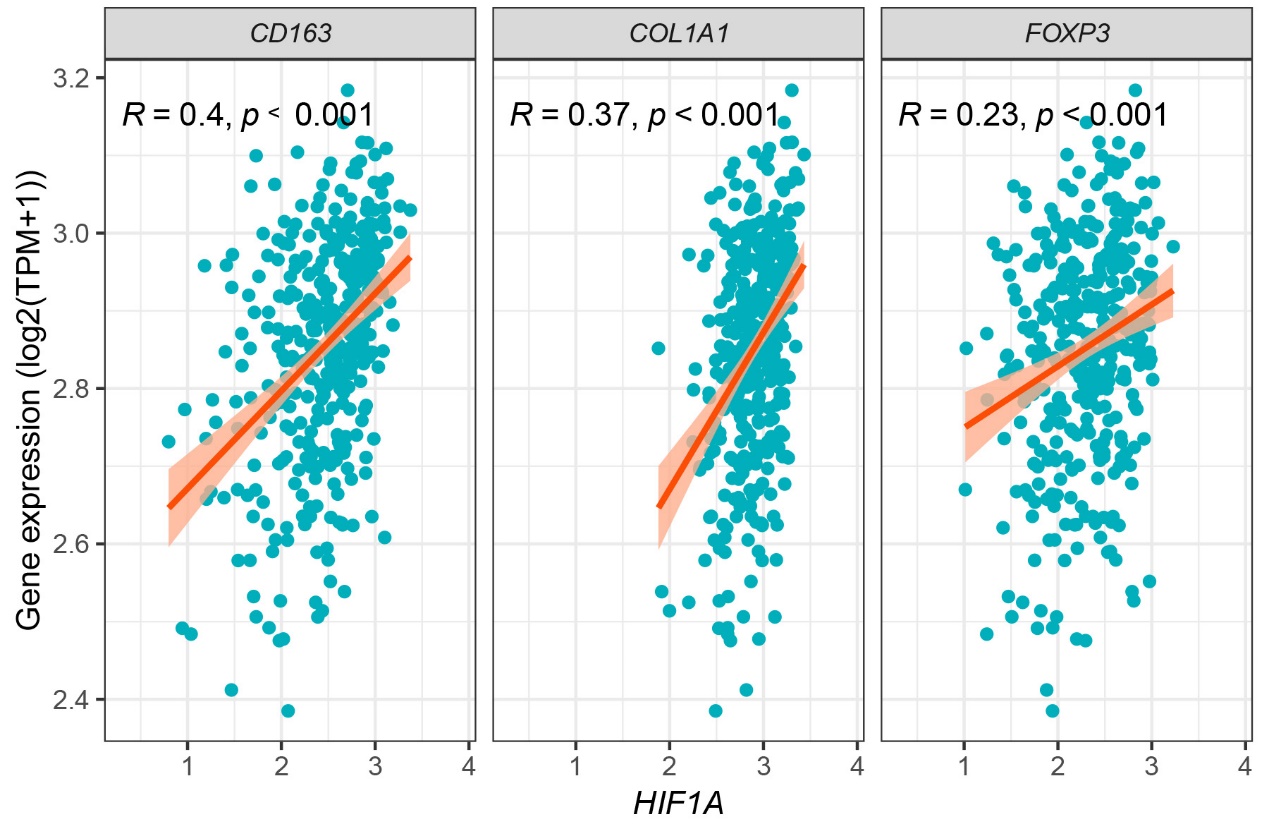


Figure S1. Correlation analysis of *HIF1A* with *CD163*, *COL1A1*, and *FOXP3*.


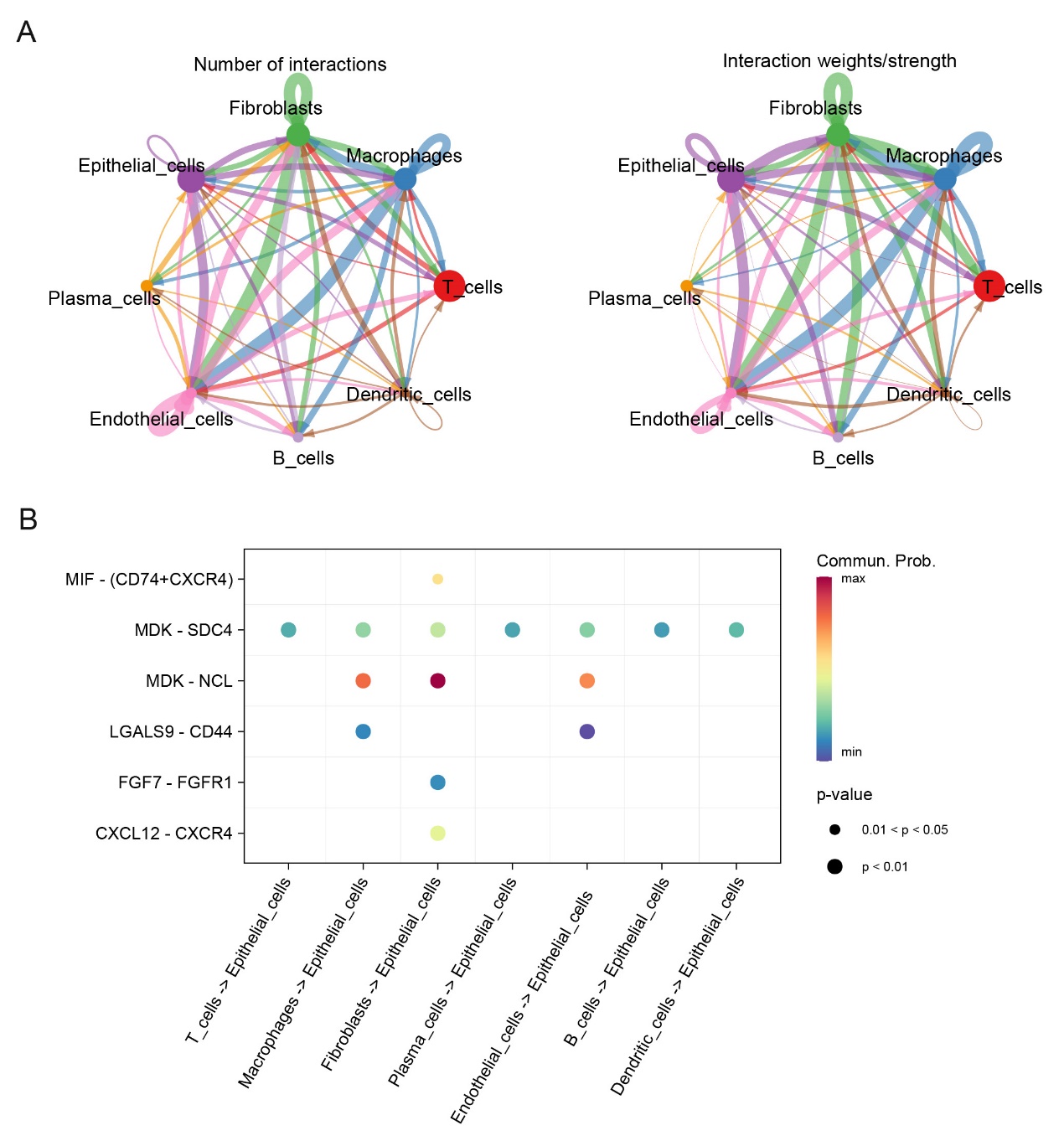


Figure S2. (A) The number and weight of cell-cell interactions. (B) Ligand-receptor analysis between epithelial cells and other cells.
